# 4-(Adamantan-1-yl)-2-(4-fluoro­phen­yl)quinoline

**DOI:** 10.1107/S1600536813012336

**Published:** 2013-05-15

**Authors:** Zuzana Kozubková, Eva Babjaková, Peter Bartoš, Robert Vícha

**Affiliations:** a Department of Chemistry, Faculty of Technology, Tomas Bata University in Zlin, Nám. T. G. Masaryka 275, Zlín, 762 72, Czech Republic; b Department of Chemistry, Faculty of Science, Masaryk University, Kamenice 5, Brno-Bohunice, 625 00, Czech Republic

## Abstract

In the mol­ecule of the title compound, C_25_H_24_FN, the dihedral angle between the best planes of the quinoline fragment (rings *A* and *B*) and the benzene ring (*C*) is 9.51 (4)°. In the crystal, mol­ecules are linked into centrosymmetric dimers *via* pairs of weak C—H⋯F inter­actions. The mol­ecules are stacked into chains along the *a* axis by weak off-set π–π inter­actions between the *A* and *C* rings of translation-related mol­ecules with a centroid–centroid distance of 3.6440 (2) Å.

## Related literature

For the preparation and spectroscopic properties of the title compound, see: Kozubková *et al.* (2012 ▶). For related structures, see: Kozubková *et al.* (2012 ▶); Prabhuswamy *et al.* (2012 ▶). For the biological activity of related compounds, see: Nayyar *et al.* (2009 ▶).
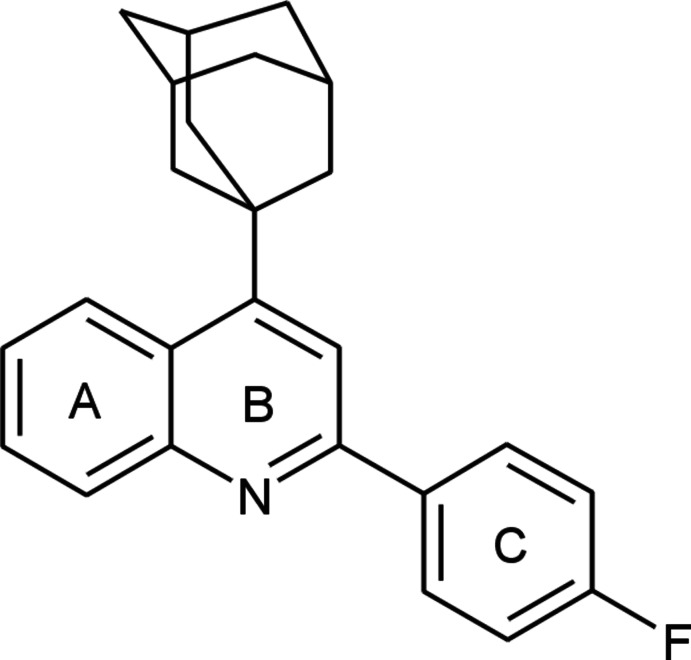


## Experimental

### 

#### Crystal data

C_25_H_24_FN
*M* *_r_* = 357.45
Triclinic, 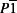

*a* = 6.4604 (3) Å
*b* = 10.9964 (4) Å
*c* = 12.9074 (5) Å
α = 93.205 (3)°
β = 96.446 (3)°
γ = 100.507 (3)°
*V* = 893.14 (6) Å^3^
*Z* = 2
Mo *K*α radiation
μ = 0.08 mm^−1^
*T* = 120 K
0.60 × 0.40 × 0.20 mm

#### Data collection

Oxford Diffraction Xcalibur diffractometer
Absorption correction: multi-scan (*CrysAlis RED*; Oxford Diffraction, 2009 ▶) *T*_min_ = 0.971, *T*_max_ = 1.000
9490 measured reflections
3142 independent reflections
2445 reflections with *I* > 2σ(*I*)
*R*_int_ = 0.015

#### Refinement

*R*[*F* ^2^ > 2σ(*F* ^2^)] = 0.034
*wR*(*F* ^2^) = 0.099
*S* = 1.08
3142 reflections
244 parameters
H-atom parameters constrained
Δρ_max_ = 0.20 e Å^−3^
Δρ_min_ = −0.19 e Å^−3^

### 

Data collection: *CrysAlis CCD* (Oxford Diffraction, 2009 ▶); cell refinement: *CrysAlis RED* (Oxford Diffraction, 2009 ▶); data reduction: *CrysAlis RED*; program(s) used to solve structure: *SHELXS97* (Sheldrick, 2008 ▶); program(s) used to refine structure: *SHELXL97* (Sheldrick, 2008 ▶); molecular graphics: *ORTEP-3 for Windows* (Farrugia, 2012 ▶) and *Mercury* (Macrae *et al.*, 2008 ▶); software used to prepare material for publication: *SHELXL97*.

## Supplementary Material

Crystal structure: contains datablock(s) global, I. DOI: 10.1107/S1600536813012336/fy2093sup1.cif

Structure factors: contains datablock(s) I. DOI: 10.1107/S1600536813012336/fy2093Isup2.hkl

Supplementary material file. DOI: 10.1107/S1600536813012336/fy2093Isup3.cml

Additional supplementary materials:  crystallographic information; 3D view; checkCIF report

## Figures and Tables

**Table 1:** Hydrogen-bond geometry (Å, °)

*D*—H⋯*A*	*D*—H	H⋯*A*	*D*⋯*A*	*D*—H⋯*A*
C12—H12⋯F1^i^	0.95	2.61	3.2955 (12)	129

Symmetry code: (i) 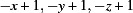.

**Fig. 1.**
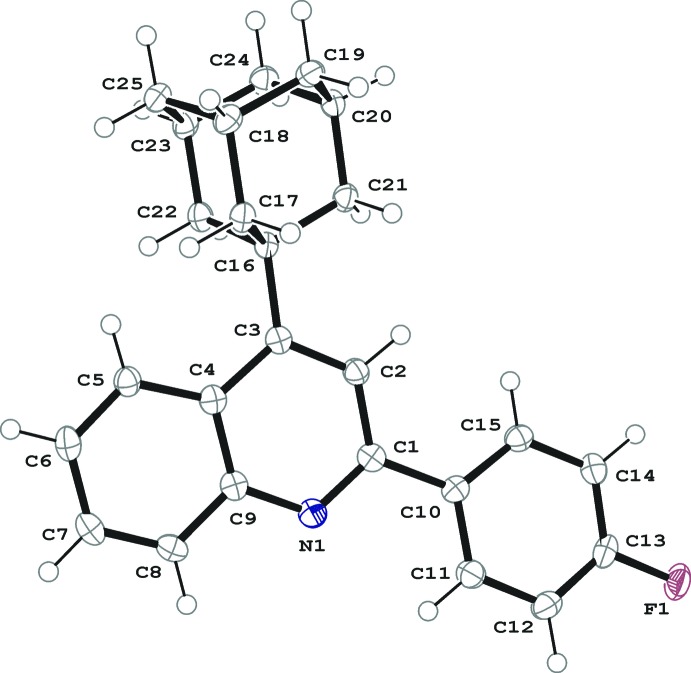
ORTEP view of the asymmetric unit with atoms represented as 50% probability ellipsoids. H-atoms are shown as small spheres at arbitrary radii.

**Fig. 2.**
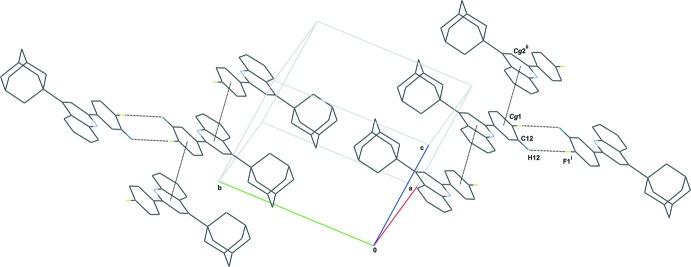
Part of the crystal structure of the title compound showing the intermolecular π–π and C–H···F interactions as dotted lines. H-atoms have been omitted for clarity (except for those participating in H-bonds). Cg1 and Cg2 are the respective centers of gravity of the C10–C15 and C1–C4,C9,N1 rings. Symmetry codes: (i) -x + 1, -y + 1, -z + 1; (ii) x - 1, y, z.

## supplementary crystallographic information

### Comment

Quinoline and its derivatives are very important compounds whose structures occur in a variety of natural alkaloids and therapeutics with interesting biological activities. As an example, derivatives of 4-(1-adamantyl)quinoline-2-carboxylic acid have been recently described as potent antituberculosis agents (Nayyar *et al.*, 2009). The title compound was prepared as a part of our research aimed at the examination of novel convenient synthetic procedures toward 4-(1-adamantyl)quinoline derivatives (Kozubková *et al.*, 2012).

The molecule of the title compound consists of three motifs – quinoline and benzene rings and adamantane cage (Figure 1). The quinoline and benzene rings are essentially planar with the respective r.m.s. deviations of 0.0303 and 0.0052 Å and the respective maximum deviations of -0.0477 (12) Å for C1 and 0.0067 (12) Å for C13. The dihedral angle between the planes of these rings is 9.51 (4)°. The adamantane cage consist of free fused cyclohexane rings in classical chairs conformations with C—C—C angles in the range 105.68 (10)–111.83 (11)°. The torsion angles describing the mutual orientation of the benzene, quinoline and adamantane moieties C2–C1–C10–C15 and C4–C3–C16–C17 are -10.62 (19) and 66.77 (15)°, respectively. Although the quinoline ring is essentially planar, it is markedly deformed in plane as it is usual for similar quinoline rings substituted with bulky adamantane at position 4 (Kozubková *et al.*, 2012). The most affected valence angles N1–C9–C8, C2–C3–C4, N1–C9–C4 and C3–C4–C5 are 115.82 (11), 115.91 (11), 124.06 (12) and 125.91 (12)°, respectively. The packing of the molecules in the crystal is stabilized by a weak C—H···F interaction (Table 1, Figure 2). In addition, a weak π–π interaction (Figure 2) is observed between the benzene ring (C10–C15) and quinoline ring (C1–C9, N1), with the shortest centroid-to-centroid distance of 3.6440 (2) Å. Distances of C14, C15, and *Cg*1 from best plane of adjacent quinoline ring are -3.2742 (13), -3.2448 (13), and -3.4517 (13) Å.

### Experimental

The title compound was prepared *via* a Friedländer reaction from the corresponding 1-adamantyl aminophenyl ketone and 4-fluoroacetophenone as it has been described previously (Kozubková *et al.*, 2012). A single-crystal usable for X-ray analysis was obtained by slow spontaneous evaporation from deuterochloloform at room temperature.

### Refinement

All H atoms were placed at calculated positions and were refined as riding with their *U*_iso_ set to 1.2Ueq of the respective carrier atoms. C—H distances are 0.9500 Å for aromatic H atoms, 0.9900 Å for H atoms of secondary carbon atoms and 1.0000 Å for H atoms of tertiary carbon atoms.

### Crystal data

**Table:**

C_25_H_24_FN	*Z* = 2
*M**_r_* = 357.45	*F*(000) = 380
Triclinic, *P*1	*D*_x_ = 1.329 Mg m^−^^3^
Hall symbol: -P 1	Melting point: 435 K
*a* = 6.4604 (3) Å	Mo *K*α radiation, λ = 0.71073 Å
*b* = 10.9964 (4) Å	Cell parameters from 5109 reflections
*c* = 12.9074 (5) Å	θ = 3.2–27.2°
α = 93.205 (3)°	µ = 0.08 mm^−^^1^
β = 96.446 (3)°	*T* = 120 K
γ = 100.507 (3)°	Block, colourless
*V* = 893.14 (6) Å^3^	0.60 × 0.40 × 0.20 mm

### Data collection

**Table:**

Oxford Diffraction Xcalibur diffractometer	3142 independent reflections
Radiation source: Enhance (Mo) X-ray Source	2445 reflections with *I* > 2σ(*I*)
Graphite monochromator	*R*_int_ = 0.015
Detector resolution: 8.4353 pixels mm^-1^	θ_max_ = 25.0°, θ_min_ = 3.2°
ω scan	*h* = −7→7
Absorption correction: multi-scan (*CrysAlis RED*; Oxford Diffraction, 2009)	*k* = −8→13
*T*_min_ = 0.971, *T*_max_ = 1.000	*l* = −15→15
9490 measured reflections	

### Refinement

**Table:**

Refinement on *F*^2^	0 restraints
Least-squares matrix: full	H-atom parameters constrained
*R*[*F*^2^ > 2σ(*F*^2^)] = 0.034	*w* = 1/[σ^2^(*F*_o_^2^) + (0.0613*P*)^2^] where *P* = (*F*_o_^2^ + 2*F*_c_^2^)/3
*wR*(*F*^2^) = 0.099	(Δ/σ)_max_ < 0.001
*S* = 1.08	Δρ_max_ = 0.20 e Å^−^^3^
3142 reflections	Δρ_min_ = −0.19 e Å^−^^3^
244 parameters	

### Special details

**Table:**

Geometry. All e.s.d.'s (except the e.s.d. in the dihedral angle between two l.s. planes) are estimated using the full covariance matrix. The cell e.s.d.'s are taken into account individually in the estimation of e.s.d.'s in distances, angles and torsion angles; correlations between e.s.d.'s in cell parameters are only used when they are defined by crystal symmetry. An approximate (isotropic) treatment of cell e.s.d.'s is used for estimating e.s.d.'s involving l.s. planes.
Refinement. Refinement of *F*^2^ against ALL reflections. The weighted *R*-factor *wR* and goodness of fit *S* are based on *F*^2^, conventional *R*-factors *R* are based on *F*, with *F* set to zero for negative *F*^2^. The threshold expression of *F*^2^ > 2σ(*F*^2^) is used only for calculating *R*-factors(gt) *etc*. and is not relevant to the choice of reflections for refinement. *R*-factors based on *F*^2^ are statistically about twice as large as those based on *F*, and *R*- factors based on ALL data will be even larger.

### Fractional atomic coordinates and isotropic or equivalent isotropic displacement parameters (Å^2^)

**Table:**

	*x*	*y*	*z*	*U*_iso_*/*U*_eq_	
F1	0.34394 (12)	0.33553 (7)	0.56712 (6)	0.0332 (2)	
N1	1.06928 (16)	0.26583 (10)	0.29611 (8)	0.0214 (3)	
C1	0.92455 (19)	0.17633 (12)	0.32331 (9)	0.0198 (3)	
C2	0.91382 (19)	0.05025 (12)	0.29032 (9)	0.0200 (3)	
H2	0.8044	−0.0101	0.3110	0.024*	
C3	1.05410 (19)	0.01109 (12)	0.22984 (9)	0.0187 (3)	
C4	1.21873 (19)	0.10663 (12)	0.20249 (9)	0.0194 (3)	
C5	1.39056 (19)	0.08736 (13)	0.14764 (9)	0.0213 (3)	
H5	1.4001	0.0052	0.1247	0.026*	
C6	1.5421 (2)	0.18375 (13)	0.12701 (9)	0.0243 (3)	
H6	1.6546	0.1674	0.0902	0.029*	
C7	1.5339 (2)	0.30672 (13)	0.15946 (10)	0.0261 (3)	
H7	1.6392	0.3733	0.1441	0.031*	
C8	1.3735 (2)	0.32951 (13)	0.21319 (10)	0.0244 (3)	
H8	1.3678	0.4126	0.2351	0.029*	
C9	1.21530 (19)	0.23188 (12)	0.23691 (9)	0.0202 (3)	
C10	0.77164 (19)	0.21533 (12)	0.39064 (9)	0.0195 (3)	
C11	0.8057 (2)	0.33784 (12)	0.43399 (10)	0.0236 (3)	
H11	0.9288	0.3944	0.4218	0.028*	
C12	0.6640 (2)	0.37833 (13)	0.49428 (10)	0.0250 (3)	
H12	0.6889	0.4615	0.5240	0.030*	
C13	0.4865 (2)	0.29515 (13)	0.51007 (9)	0.0236 (3)	
C14	0.4466 (2)	0.17378 (13)	0.47036 (9)	0.0237 (3)	
H14	0.3231	0.1181	0.4834	0.028*	
C15	0.59069 (19)	0.13429 (12)	0.41086 (9)	0.0216 (3)	
H15	0.5658	0.0503	0.3832	0.026*	
C16	1.03604 (19)	−0.12759 (12)	0.19886 (9)	0.0180 (3)	
C17	1.22648 (19)	−0.17477 (12)	0.25635 (9)	0.0197 (3)	
H17A	1.3607	−0.1264	0.2383	0.024*	
H17B	1.2260	−0.1621	0.3329	0.024*	
C18	1.2147 (2)	−0.31265 (12)	0.22571 (9)	0.0218 (3)	
H18	1.3411	−0.3403	0.2620	0.026*	
C19	1.0124 (2)	−0.38832 (12)	0.25789 (9)	0.0230 (3)	
H19A	1.0050	−0.4776	0.2390	0.028*	
H19B	1.0131	−0.3763	0.3345	0.028*	
C20	0.82066 (19)	−0.34565 (12)	0.20168 (9)	0.0214 (3)	
H20	0.6879	−0.3938	0.2235	0.026*	
C21	0.83436 (19)	−0.20723 (12)	0.23111 (9)	0.0203 (3)	
H21A	0.8346	−0.1938	0.3076	0.024*	
H21B	0.7076	−0.1804	0.1963	0.024*	
C22	1.0223 (2)	−0.15445 (12)	0.07863 (9)	0.0207 (3)	
H22A	0.8924	−0.1307	0.0441	0.025*	
H22B	1.1463	−0.1039	0.0528	0.025*	
C23	1.0176 (2)	−0.29224 (12)	0.05047 (9)	0.0220 (3)	
H23	1.0164	−0.3067	−0.0268	0.026*	
C24	0.8172 (2)	−0.36869 (13)	0.08344 (9)	0.0238 (3)	
H24A	0.8113	−0.4579	0.0648	0.029*	
H24B	0.6901	−0.3444	0.0465	0.029*	
C25	1.2133 (2)	−0.33189 (13)	0.10712 (9)	0.0235 (3)	
H25A	1.2110	−0.4204	0.0871	0.028*	
H25B	1.3437	−0.2822	0.0862	0.028*	

### Atomic displacement parameters (Å^2^)

**Table:**

	*U*^11^	*U*^22^	*U*^33^	*U*^12^	*U*^13^	*U*^23^
F1	0.0378 (5)	0.0358 (5)	0.0331 (4)	0.0163 (4)	0.0192 (4)	0.0045 (4)
N1	0.0203 (6)	0.0218 (7)	0.0228 (5)	0.0056 (5)	0.0024 (4)	0.0036 (5)
C1	0.0184 (7)	0.0214 (8)	0.0192 (6)	0.0043 (6)	−0.0010 (5)	0.0032 (6)
C2	0.0183 (6)	0.0196 (8)	0.0221 (6)	0.0028 (6)	0.0040 (5)	0.0026 (6)
C3	0.0176 (6)	0.0214 (8)	0.0165 (6)	0.0034 (6)	0.0001 (5)	0.0022 (5)
C4	0.0194 (6)	0.0233 (8)	0.0154 (6)	0.0043 (6)	0.0000 (5)	0.0031 (5)
C5	0.0212 (7)	0.0244 (8)	0.0180 (6)	0.0034 (6)	0.0026 (5)	0.0015 (5)
C6	0.0208 (7)	0.0325 (9)	0.0195 (6)	0.0029 (6)	0.0051 (5)	0.0041 (6)
C7	0.0235 (7)	0.0271 (9)	0.0265 (7)	−0.0007 (6)	0.0051 (6)	0.0076 (6)
C8	0.0270 (7)	0.0185 (8)	0.0271 (7)	0.0020 (6)	0.0035 (6)	0.0049 (6)
C9	0.0192 (6)	0.0230 (8)	0.0187 (6)	0.0048 (6)	0.0008 (5)	0.0041 (6)
C10	0.0204 (7)	0.0202 (8)	0.0186 (6)	0.0065 (6)	0.0007 (5)	0.0017 (5)
C11	0.0229 (7)	0.0218 (8)	0.0261 (7)	0.0034 (6)	0.0035 (5)	0.0022 (6)
C12	0.0309 (8)	0.0217 (8)	0.0235 (7)	0.0087 (6)	0.0036 (6)	−0.0004 (6)
C13	0.0268 (7)	0.0306 (9)	0.0179 (6)	0.0139 (7)	0.0068 (5)	0.0040 (6)
C14	0.0226 (7)	0.0268 (9)	0.0230 (6)	0.0052 (6)	0.0056 (5)	0.0062 (6)
C15	0.0230 (7)	0.0202 (8)	0.0219 (6)	0.0056 (6)	0.0020 (5)	0.0012 (6)
C16	0.0172 (6)	0.0187 (8)	0.0183 (6)	0.0033 (6)	0.0044 (5)	0.0010 (5)
C17	0.0174 (6)	0.0236 (8)	0.0179 (6)	0.0033 (6)	0.0030 (5)	0.0003 (5)
C18	0.0220 (7)	0.0229 (8)	0.0222 (6)	0.0086 (6)	0.0029 (5)	0.0013 (6)
C19	0.0313 (7)	0.0183 (8)	0.0212 (6)	0.0068 (6)	0.0075 (5)	0.0008 (6)
C20	0.0194 (7)	0.0209 (8)	0.0236 (6)	0.0006 (6)	0.0077 (5)	0.0005 (6)
C21	0.0178 (6)	0.0224 (8)	0.0212 (6)	0.0043 (6)	0.0048 (5)	0.0005 (6)
C22	0.0188 (6)	0.0249 (8)	0.0179 (6)	0.0022 (6)	0.0026 (5)	0.0023 (5)
C23	0.0247 (7)	0.0253 (8)	0.0155 (6)	0.0033 (6)	0.0043 (5)	−0.0023 (5)
C24	0.0230 (7)	0.0240 (8)	0.0227 (7)	0.0017 (6)	0.0019 (5)	−0.0026 (6)
C25	0.0238 (7)	0.0222 (8)	0.0256 (7)	0.0052 (6)	0.0076 (5)	−0.0018 (6)

### Geometric parameters (Å, º)

**Table:**

F1—C13	1.3610 (14)	C15—H15	0.9500
N1—C1	1.3201 (16)	C16—C21	1.5441 (16)
N1—C9	1.3681 (16)	C16—C17	1.5506 (16)
C1—C2	1.4144 (17)	C16—C22	1.5530 (15)
C1—C10	1.4901 (17)	C17—C18	1.5313 (17)
C2—C3	1.3710 (16)	C17—H17A	0.9900
C2—H2	0.9500	C17—H17B	0.9900
C3—C4	1.4414 (17)	C18—C19	1.5287 (17)
C3—C16	1.5348 (17)	C18—C25	1.5322 (16)
C4—C5	1.4227 (17)	C18—H18	1.0000
C4—C9	1.4282 (18)	C19—C20	1.5262 (17)
C5—C6	1.3654 (18)	C19—H19A	0.9900
C5—H5	0.9500	C19—H19B	0.9900
C6—C7	1.4051 (18)	C20—C24	1.5300 (16)
C6—H6	0.9500	C20—C21	1.5322 (17)
C7—C8	1.3617 (18)	C20—H20	1.0000
C7—H7	0.9500	C21—H21A	0.9900
C8—C9	1.4134 (18)	C21—H21B	0.9900
C8—H8	0.9500	C22—C23	1.5324 (17)
C10—C15	1.3941 (17)	C22—H22A	0.9900
C10—C11	1.3986 (18)	C22—H22B	0.9900
C11—C12	1.3826 (17)	C23—C24	1.5264 (17)
C11—H11	0.9500	C23—C25	1.5337 (17)
C12—C13	1.3727 (19)	C23—H23	1.0000
C12—H12	0.9500	C24—H24A	0.9900
C13—C14	1.3708 (18)	C24—H24B	0.9900
C14—C15	1.3838 (17)	C25—H25A	0.9900
C14—H14	0.9500	C25—H25B	0.9900
			
C1—N1—C9	117.34 (11)	C18—C17—H17A	109.5
N1—C1—C2	122.11 (11)	C16—C17—H17A	109.5
N1—C1—C10	116.32 (12)	C18—C17—H17B	109.5
C2—C1—C10	121.57 (11)	C16—C17—H17B	109.5
C3—C2—C1	123.02 (12)	H17A—C17—H17B	108.1
C3—C2—H2	118.5	C19—C18—C17	109.63 (10)
C1—C2—H2	118.5	C19—C18—C25	109.72 (10)
C2—C3—C4	115.91 (12)	C17—C18—C25	109.25 (10)
C2—C3—C16	120.26 (11)	C19—C18—H18	109.4
C4—C3—C16	123.79 (11)	C17—C18—H18	109.4
C5—C4—C9	116.54 (12)	C25—C18—H18	109.4
C5—C4—C3	125.90 (12)	C20—C19—C18	108.95 (10)
C9—C4—C3	117.51 (11)	C20—C19—H19A	109.9
C6—C5—C4	121.80 (13)	C18—C19—H19A	109.9
C6—C5—H5	119.1	C20—C19—H19B	109.9
C4—C5—H5	119.1	C18—C19—H19B	109.9
C5—C6—C7	120.96 (12)	H19A—C19—H19B	108.3
C5—C6—H6	119.5	C19—C20—C24	109.41 (10)
C7—C6—H6	119.5	C19—C20—C21	109.41 (10)
C8—C7—C6	119.30 (13)	C24—C20—C21	110.10 (10)
C8—C7—H7	120.4	C19—C20—H20	109.3
C6—C7—H7	120.4	C24—C20—H20	109.3
C7—C8—C9	121.27 (13)	C21—C20—H20	109.3
C7—C8—H8	119.4	C20—C21—C16	111.83 (10)
C9—C8—H8	119.4	C20—C21—H21A	109.3
N1—C9—C8	115.81 (12)	C16—C21—H21A	109.3
N1—C9—C4	124.04 (12)	C20—C21—H21B	109.3
C8—C9—C4	120.11 (11)	C16—C21—H21B	109.3
C15—C10—C11	117.95 (12)	H21A—C21—H21B	107.9
C15—C10—C1	122.31 (12)	C23—C22—C16	110.71 (10)
C11—C10—C1	119.73 (11)	C23—C22—H22A	109.5
C12—C11—C10	121.36 (12)	C16—C22—H22A	109.5
C12—C11—H11	119.3	C23—C22—H22B	109.5
C10—C11—H11	119.3	C16—C22—H22B	109.5
C13—C12—C11	118.23 (13)	H22A—C22—H22B	108.1
C13—C12—H12	120.9	C24—C23—C22	109.06 (10)
C11—C12—H12	120.9	C24—C23—C25	109.39 (10)
F1—C13—C14	118.85 (12)	C22—C23—C25	110.17 (10)
F1—C13—C12	118.38 (12)	C24—C23—H23	109.4
C14—C13—C12	122.77 (12)	C22—C23—H23	109.4
C13—C14—C15	118.37 (12)	C25—C23—H23	109.4
C13—C14—H14	120.8	C23—C24—C20	108.93 (10)
C15—C14—H14	120.8	C23—C24—H24A	109.9
C14—C15—C10	121.32 (13)	C20—C24—H24A	109.9
C14—C15—H15	119.3	C23—C24—H24B	109.9
C10—C15—H15	119.3	C20—C24—H24B	109.9
C3—C16—C21	112.40 (10)	H24A—C24—H24B	108.3
C3—C16—C17	109.61 (10)	C18—C25—C23	109.93 (10)
C21—C16—C17	106.18 (10)	C18—C25—H25A	109.7
C3—C16—C22	111.85 (10)	C23—C25—H25A	109.7
C21—C16—C22	105.68 (10)	C18—C25—H25B	109.7
C17—C16—C22	110.94 (10)	C23—C25—H25B	109.7
C18—C17—C16	110.84 (10)	H25A—C25—H25B	108.2
			
C9—N1—C1—C2	−2.15 (17)	C11—C10—C15—C14	1.14 (18)
C9—N1—C1—C10	178.09 (10)	C1—C10—C15—C14	−177.50 (11)
N1—C1—C2—C3	1.36 (18)	C2—C3—C16—C21	7.19 (15)
C10—C1—C2—C3	−178.88 (11)	C4—C3—C16—C21	−175.40 (10)
C1—C2—C3—C4	1.05 (17)	C2—C3—C16—C17	−110.64 (12)
C1—C2—C3—C16	178.66 (10)	C4—C3—C16—C17	66.78 (13)
C2—C3—C4—C5	174.85 (11)	C2—C3—C16—C22	125.88 (12)
C16—C3—C4—C5	−2.67 (18)	C4—C3—C16—C22	−56.70 (14)
C2—C3—C4—C9	−2.43 (15)	C3—C16—C17—C18	−179.14 (9)
C16—C3—C4—C9	−179.94 (10)	C21—C16—C17—C18	59.23 (12)
C9—C4—C5—C6	−1.31 (17)	C22—C16—C17—C18	−55.13 (13)
C3—C4—C5—C6	−178.61 (11)	C16—C17—C18—C19	−61.68 (12)
C4—C5—C6—C7	−0.01 (18)	C16—C17—C18—C25	58.58 (12)
C5—C6—C7—C8	0.69 (18)	C17—C18—C19—C20	60.25 (12)
C6—C7—C8—C9	0.01 (18)	C25—C18—C19—C20	−59.73 (13)
C1—N1—C9—C8	−177.26 (10)	C18—C19—C20—C24	61.41 (13)
C1—N1—C9—C4	0.59 (17)	C18—C19—C20—C21	−59.29 (12)
C7—C8—C9—N1	176.56 (11)	C19—C20—C21—C16	60.65 (12)
C7—C8—C9—C4	−1.39 (18)	C24—C20—C21—C16	−59.63 (13)
C5—C4—C9—N1	−175.79 (10)	C3—C16—C21—C20	−178.86 (9)
C3—C4—C9—N1	1.74 (17)	C17—C16—C21—C20	−59.02 (12)
C5—C4—C9—C8	1.98 (16)	C22—C16—C21—C20	58.88 (12)
C3—C4—C9—C8	179.51 (10)	C3—C16—C22—C23	176.68 (10)
N1—C1—C10—C15	169.13 (10)	C21—C16—C22—C23	−60.71 (12)
C2—C1—C10—C15	−10.64 (18)	C17—C16—C22—C23	53.95 (13)
N1—C1—C10—C11	−9.49 (16)	C16—C22—C23—C24	63.30 (12)
C2—C1—C10—C11	170.75 (10)	C16—C22—C23—C25	−56.77 (13)
C15—C10—C11—C12	−0.61 (18)	C22—C23—C24—C20	−60.27 (13)
C1—C10—C11—C12	178.06 (11)	C25—C23—C24—C20	60.28 (14)
C10—C11—C12—C13	−0.57 (19)	C19—C20—C24—C23	−61.85 (14)
C11—C12—C13—F1	−178.33 (11)	C21—C20—C24—C23	58.43 (13)
C11—C12—C13—C14	1.30 (19)	C19—C18—C25—C23	58.80 (14)
F1—C13—C14—C15	178.84 (11)	C17—C18—C25—C23	−61.41 (13)
C12—C13—C14—C15	−0.79 (19)	C24—C23—C25—C18	−59.05 (14)
C13—C14—C15—C10	−0.47 (18)	C22—C23—C25—C18	60.83 (13)

### Hydrogen-bond geometry (Å, º)

**Table:**

*D*—H···*A*	*D*—H	H···*A*	*D*···*A*	*D*—H···*A*
C12—H12···F1^i^	0.95	2.61	3.2955 (12)	129

Symmetry code: (i) −*x*+1, −*y*+1, −*z*+1.
